# Author Correction: Federated deep learning for detecting COVID-19 lung abnormalities in CT: a privacy-preserving multinational validation study

**DOI:** 10.1038/s41746-022-00600-1

**Published:** 2022-04-24

**Authors:** Qi Dou, Tiffany Y. So, Meirui Jiang, Quande Liu, Varut Vardhanabhuti, Georgios Kaissis, Zeju Li, Weixin Si, Heather H. C. Lee, Kevin Yu, Zuxin Feng, Li Dong, Egon Burian, Friederike Jungmann, Rickmer Braren, Marcus Makowski, Bernhard Kainz, Daniel Rueckert, Ben Glocker, Simon C. H. Yu, Pheng Ann Heng

**Affiliations:** 1 Department of Computer Science and Engineering, The Chinese University of Hong Kong, Hong Kong SAR, China; 2 Department of Imaging and Interventional Radiology, The Chinese University of Hong Kong, Hong Kong SAR, China; 3 Department of Diagnostic Radiology, Li Ka Shing Faculty of Medicine, The University of Hong Kong, Hong Kong SAR, China; 4 Biomedical Image Analysis Group, Imperial College London, London, UK; 5 Institute for Diagnostic and Interventional Radiology, Technical University of Munich, School of Medicine, Munich, Germany; 6 OpenMined, Oxford, UK; 7 Shenzhen Institutes of Advanced Technology, Chinese Academy of Sciences, Shenzhen, Guangdong China; 8 Department of Diagnostic Radiology, Princess Margaret Hospital, Hong Kong SAR, China; 9 Department of Radiology, Tuen Muen Hospital, Hong Kong SAR, China; 10 Department of Emergency Medicine, Peking University ShenZhen Hospital, Shenzhen, Guangdong China; 11 Department of Radiology, Zhijiang People’s Hospital, Zhijiang, Hubei China; 12 German Cancer Research Center (DKFZ), Heidelberg, Germany; 13 AI in Medicine and Healthcare, Technical University of Munich, School of Informatics and Medicine, Munich, Germany

**Keywords:** Diagnostic markers, Computed tomography

Correction to: *npj Digital Medicine* 10.1038/s41746-021-00431-6, published online 29 March 2021

In this Article the affiliation details for Egon Burian, Friederike Jungmann, Rickmer Braren, and Marcus Makowski were incorrectly given as ‘Department of Emergency Medicine, Peking University ShenZhen Hospital, Shenzhen, Guangdong, China’ but should have been ‘Institute for Diagnostic and Interventional Radiology, Technical University of Munich, School of Medicine, Munich, Germany’. The original article has been corrected.

